# Molecular investigation of Feline Panleukopenia in an endangered leopard (Panthera pardus) – a case report

**DOI:** 10.1186/s12917-023-03612-5

**Published:** 2023-03-02

**Authors:** S. M. Kolangath, S. V. Upadhye, V. M. Dhoot, M. D. Pawshe, B. K. Bhadane, A. P. Gawande, R. M. Kolangath

**Affiliations:** 1 Wildlife Research & Training Centre, MAFSU, Nagpur, Opp. Hindustan Lever Godown Square, Maharashtra Animal & Fishery Sciences University, Mahurzhari Road, Fetri, 441501 Nagpur, India; 2 Department of Biotechnology & Biochemistry, Saint Francis DeSales College, Seminary Hills, 440006 Nagpur, India

**Keywords:** Leopard, *Panthera pardus*, Feline panleukopenia, Feline panleukopenia virus (FPLV), Wildlife, Parvovirus

## Abstract

**Background:**

Feline Panleukopenia is an important disease of cats and has been reported worldwide. The disease is caused by a non-enveloped, single-stranded DNA virus; Feline Panleukopenia Virus (FPLV), belonging to the Parvoviridae family. The disease causes significant mortality in unvaccinated kittens. The disease has been well documented in companion animals. However, only a few reports have surfaced from the wild.

**Case presentation:**

An orphan leopard cub was presented to Wildlife Rescue Centre, Nagpur, for further care; the leopard was kept under quarantine. On day 22 of the quarantine, the leopard showed inappetence, lethargy and depression and did not consume the offered carabeef (Day 0 of treatment). The leopard was examined clinically and was found to have a temperature of 102°F; blood was collected and analysed. On day one, the leopard exhibited bloody diarrhoea, inappetence, fever and depression. The leopard was rationally treated with fluids, antibiotics, multi-vitamins, haemostatics and haematinics. To gain qualitative insights into the epidemiological aspect of the disease, molecular investigation, including Polymerase Chain Reaction (PCR) and qPCR (Quantitative Polymerase Chain Reaction), were utilized to confirm the infection. The amplicon was sequenced and was found to be similar to sequences of FPLV reported domestic cats and other wild felids from India and abroad. Phylogenetic analysis was performed to understand the evolutionary relationship of the virus with previously reported sequences of FPLV. Sequences were submitted to National Center for Biotechnology Information (NCBI) and were allotted accession numbers.

**Conclusion:**

The infection in endangered leopard cubs could be managed with prompt fluid therapy, antibiotics and support treatment, ensuring an uneventful recovery. Molecular investigation and sequencing efforts can provide valuable data on epidemiology and the evolutionary relationship of the virus with the circulating strains in the field. The study has implications in the preventive management of FPLV in captivity and the selection of strains for inclusion in vaccines meant for the wild felids.

## Background

Feline Panleukopenia is an infectious disease of cats caused by a non-enveloped, single-stranded DNA virus; Feline Panleukopenia Virus (FPLV), belonging to the Parvoviridae family. The infection has been well documented in domestic, stray and wild cats worldwide [1, 2]. The disease is characterized by severe enteritis, dehydration and lymphopenia; the mortality in kittens ranges from 25–90% in unvaccinated cases [3]. The genome encodes for two structural proteins, VP1 and VP2, and two non-structural proteins, NS1 and NS2 respectively. VP2 region is considered crucial as it influences the viral pathogenicity, immune response and host range. The FPLV is closely related to Mink Parvovirus (MPV) and Canine Parvovirus (CPV-2), which can be attributed to local mutations in the VP2 gene of the virus. The CPV-2 has evolved from FPLV and it can infect cats and even produce clinical disease as cross-protection is weak [4]. The virus can be transmitted to a susceptible host owing to its non-enveloped nature and resistance to chemical and physical factors, which ensures viability for months in the environment [5]. An infected animal may shed 10^9^ TCID_50_/gram of faeces; which is an important source of infection. The mode of transmission is primarily by the faeco-oral route. Lymphopenia is a hallmark finding during the blood investigation of the affected animal. Few reports of intrauterine or prenatal infection in kittens leading to feline ataxia syndrome have been reported [6]. Apart from felids, FPLV is also known to infect racoons, minks and a few canid species [7]. The reports of the incidences of FPLV in the wild have been limited to tigers (*Panthera tigris*) [8], lions (*Panthera leo*) [8]; wild cats [9]; leopard cats (*Felis bengalensis*) [10]; formosan gem-faced civets (*Paguma larvata taivana*) [10]; wild fishing cat (*Prionailurus viverrinus*) [11]; wild guignas (*Leopardus guigna*) [12] serval (*Leptailurus serval*) [13]. Death due to FPLV has been reported in ten-month-old tiger and an eleven-month-old african lion cub [8]. Recent serological studies have highlighted the high prevalence of antibodies against FPLV among wild felids at the domestic animal–wildlife interface [14]. The role of passive immunity is critical in the incidence of new infection in felid neonates as maternal immunity against FPLV is known to persist till 6–8 weeks of age and plays a decisive role in the vaccination of the kittens. Vaccination is effective in controlling FPLV in companion animals; however, the nature, limited cross-protection and availability of wild-type vaccines is crucial in the containment of this significant infectious disease in wild cats.

## Case presentation

A three months old orphan leopard cub (*Panthera pardus*) was rescued from the Wadsa forest division, Gadchiroli district of Maharashtra state of India on 16^th^ September 2021. The cub was separated from its mother and reunion efforts of the forest officials failed after which the cub shifted to Transit Treatment Centre, Nagpur, on 20^th^ September 2021. After spending approximately six months at the Transit Treatment Centre the cub was shifted to Wildlife Rescue Centre, Gorewada, Nagpur, on 10^th^ March 2022. The leopard was presented to Wildlife Research & Training Centre, Nagpur, for quarantine before the introduction to the main facility. On day 22 of the quarantine, the leopard showed inappetence, lethargy and depression and did not consume the offered carabeef (Day 0 of treatment). The leopard was examined clinically and was found to have a temperature of 102°F; blood was collected using squeeze cage restraint from the coccygeal vein and analysed. On day 1, the leopard exhibited bloody diarrhoea, inappetence, fever and depression (Table 1). The complete blood count indicated leucocytopenia, thrombocytopenia and altered blood parameters; blood chemistry revealed increased creatinine. The animal was physically restrained in a squeeze cage and therapy in the form of injection Amoxycillin-Clavulanate 500 mg, injection Ringers Lactate 500 ml, injection Normal Saline 500 ml, injection Vitamin B_1_, B_6_, and B_12_ were administered intravenous along with injection Sylate. Injection Fligrastim® containing recombinant human granulocyte colony stimulating factor (rHGSF) was administered subcutaneously to correct the leukopenia. The animal was offered water at all times and kept under CCTV (Closed Circuit Television) surveillance. The low leucocyte count improved from 2900 on day 0 to 77,200 on day 6. The serum values showed a slight increase in the liver function values (Table 2), as indicated by previous reports [15, 16]. The leopard was monitored during the entire tenure of the treatment and the details of the clinical and behavioural observations were noted (Table 1). On day four, the leopard began to feed on soft meat and returned to complete appetence by Day 7. The blood parameters and serum values showed improvement during the recovery phase. The onset of the infection could be attributed to captive and transport stress, age and presence of older inmates at the quarantine facility.

**Table 1:** Timeline of clinical and behavioural observations during the treatment from day 0 to day 10

Sr. No	Day	Feeding	Vomition	Urination	Defecation	General Behaviour
1	Day 0	No	No	Unnoticed	Unnoticed	dull and depressed
2	Day 1	No	Yes (3 episodes)	Unnoticed	Watery, mucus present, with foetid smell	bloody diarrhoea, inappetence, fever, dull and depressed
3	Day 2	No Consumed approx.300 ml water	Yes (1 episodes)	Noticed	Watery, dark brown, with foetid smell	Weak, dehydrated, fever, dull and depressed. Found resting majority of the time
4	Day 3	No	Yes, Yellow coloured scanty vomitus passed	Unnoticed	Scanty faeces passed, Mucus laden	Weak, dehydrated, dull and depressed
5	Day 4	Yes, 100 gm boneless chicken consumed. Consumed 1.2 Lit water	No	Noticed	No faeces passed	dull and depressed
6	Day 5	Yes, 300 gm boneless chicken fed through forced feeding	No	Noticed	No faeces passed	dull and depressed
7	Day 6	Voluntarily consumed 300 gms boneless chicken, and consumed 1.2 Lit water	No	Noticed	Pasty brown coloured faeces passed	Found moving in the treatment cage. Alert but sluggish in activity
8	Day 7	Consumed 500 gms boneless chicken, and consumed 1.2 Lit water	No	Noticed	No faeces passed	Found moving in the treatment cage. Alert but sluggish in activity
9	Day 8	Consumed 500 gms boneless chicken, and consumed 800 ml water	No	Noticed	Pasty brown coloured faeces passed	Found moving in the treatment cage. Alert but sluggish in activity
10	Day 9	Consumed 1.3 Kgs boneless chicken, and consumed 1.4 Lit water	No	Noticed	No faeces passed	Alert, Restoration of normal activities with constant movement in the cage
11	Day 10	Consumed 800 gms boneless chicken, and consumed 1.2 Lit water	No	Noticed	No faeces passed	Alert and Active

**Table 2:** Haemato-biochemical values and peripheral smear investigation reports on day 0, 3 and 6 along with the reference value

Sr. No	Parameter	Day 0 (01/03/2022)	Day 3 (04/03/2022)	Day 6 (07/03/2022)	Reference Value
1	Lymphocytes (%)	35	32	22	12–30
2	Monocytes (%)	02	04	02	3–10
3	Neutrophils (%)	60	60	73	60–70
4	Eosinophils (%)	03	04	03	2–10
5	Basophils (%)	00	00	00	0–1
6	WBC (cmm)	2900	13500	77500	4000–10000
7	RBC (Mil/cmm)	9.44	7.78	7.67	4.2 -5.5 (Mil/cmm)
8	Platelets (lakhs/cmm)	1.41	0.52	1.27	200–500 (lakhs/cmm)
9	Haemoglobin (g/dL)	15.4	12.5	12.4	12–18
10	Mean Corpuscular Volume (MCV) (fL)	52	51	52	58–79
11	Packed Cell Volume (PCV) (%)	49.1	40	40	25–45
12	Mean Concentration Haemoglobin (MCH) (pg)	16.3	16.1	16.2	19.5–24.5
13	MCHC (g/dl)	16.3	31.3	31.0	32–36
14	BUN (mg/dL)	43	46	32	7–25
15	Creatinine (mg/dL)	1.64	1.43	1.29	0.3–1.4
16	ALT (U/L)	38	43	41	10–118
17	AST (U/L)	42	39	42	14–45
18	Peripheral Smear	No blood parasite found	No blood parasite found	No blood parasite found	––––

**Fig. 1:**
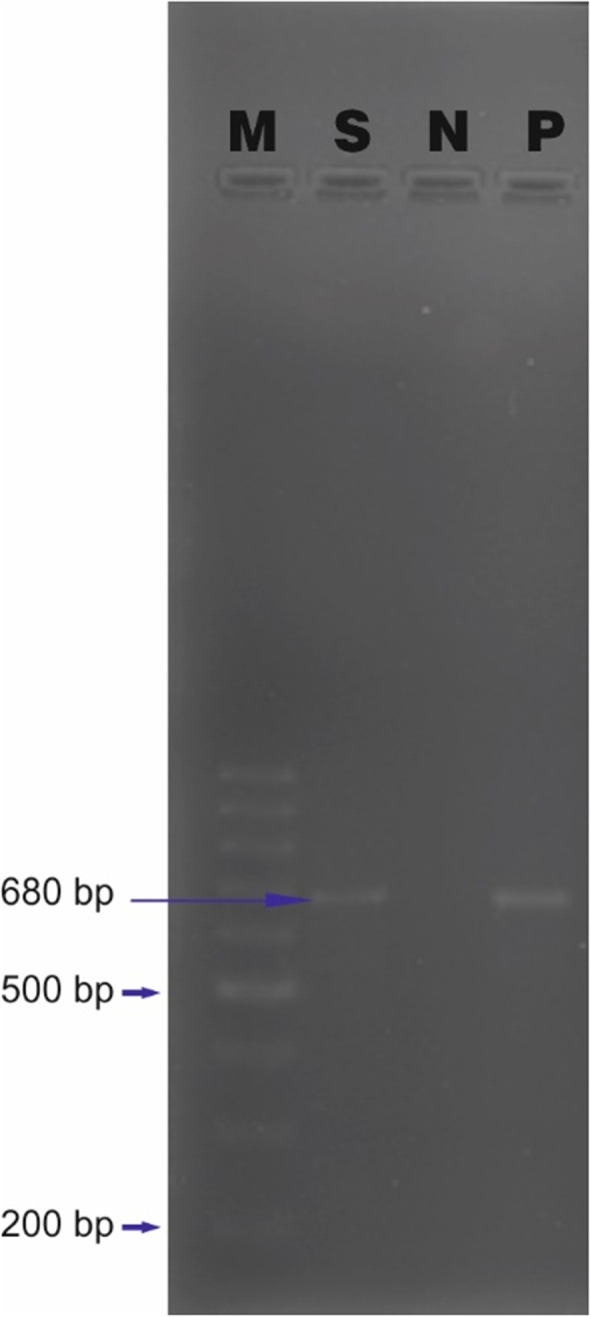
Gel electrophoresis on 1% agarose gel stained with ethidium bromide. Lane M: 100 bp ladder, Lane S: Sample, Lane N: Negative Control, Lane P: Positive Control (Canine Parvovirus-2 (CPV-2) was used as a positive control. Amplification of 680 bp obtained using and pCPV-2FP and pCPV-2RP primer

To further molecularly characterize the infection, DNA was isolated from the stool sample as per the manufacturer’s guidelines using QIAamp® Fast DNA Stool Mini Kits (Mfg. Qiagen Inc, MD, USA). A PCR targeting the VP2 region was performed using the primers CPV-2FP 5’-GAAGAGTGGTTGTAAATAATA-3’ and Pcpv-2RP 5’-CCTATATCACCAAAGTTAGTAG-3’ as per the cycling conditions mentioned in the reference [17]. An amplification of approximately 680 bp was obtained (Fig. 1), the amplicon was sequenced using the forward and reverse primers on ABI 3130 automated DNA sequencer (Mfg. Applied Biosystems, CA, USA). Canine parvovirus was used as a positive control (Accession No. OM100572), and negative control was devoid of DNA. A qPCR was also conducted to verify and quantitate the quantum of infection. Primers pCPV-2RTF 5’-CATTGGGCTTACCACCATTT-3’ and pCPV-2RTF 5’-CCAACCTCAGCTGGTCTCAT-3’ were utilized as previously described [18]. To undertake the phylogenetic analysis, similar sequences reported from domestic and wild cats from India and abroad were preferentially included in the study (Table 3). Mega XI software was used to undertake the phylogenetic analysis by the Maximum Likelihood (ML) method using bootstrap values with 1000 replications to ensure tree reliability [19].

**Table 3:** List of sequences along with their attributes utilized for the neighbour-joining phylogenetic studies

Sr. No	Accession No	Organism	Host	Country	Author
1	MK266791	Feline parvovirus	Cat	China	Unpublished
2	EU697387	Feline panleukopenia virus	Tiger	China	Unpublished
3	MT857277	Feline panleukopenia virus	Cat	Vietnam	Unpublished
4	MT857285	Feline panleukopenia virus	Cat	Vietnam	Unpublished
5	KY094112	Mink enteritis virus	Mink	China	[20]
6	MT857283	Feline panleukopenia virus	Cat	Vietnam	Unpublished
7	ON129565	Feline panleukopenia virus	Leopard	India	Unpublished
8	MK671180	Feline panleukopenia virus	Cat	China	Unpublished
9	MK671172	Feline panleukopenia virus	Cat	China	Unpublished
10	MF541122	Feline panleukopenia virus	Cat	China	Unpublished
11	KT240130	Feline panleukopenia virus	Cat	Portugal	[21]
12	KP682520	Feline panleukopenia virus	European Badger	Spain	Unpublished
13	KX900570	Feline panleukopenia virus	Jaguar (Shanghai Zoo)	China	Unpublished
14	MT078771	Feline panleukopenia virus	Domestic Cat	India	Unpublished
15	JX475256	Feline panleukopenia virus	Puma	USA	Alison et al., 2013
16	JN867594	Feline panleukopenia virus	Racoon	USA	[22]
17	EU659113	Feline panleukopenia virus	Puma	USA	[23]
18	KT240134	Feline panleukopenia virus	Domestic Cat	Portugal	[21]
19	KT240136	Feline panleukopenia virus	Domestic Cat	Portugal	[24]
20	KP033239	Feline panleukopenia virus	Cheetah	South Africa	Unpublished
21	KP033236	Feline panleukopenia virus	Cheetah	South Africa	Unpublished
22	MK052681	Feline panleukopenia virus	Cat	India	[25]
23	GU392246	Mink enteritis virus	Mink	China	Unpublished
24	KC677618	Mink enteritis virus	Fox	China	Unpublished
25	GU392256	Mink enteritis virus	Mink	China	Unpublished
26	AY665656	Mink enteritis virus	Mink	Russia	Unpublished
27	MK332007	Canine parvovirus	Dog	China	Unpublished
28	HQ883267	Canine parvovirus	Dog	China	Unpublished
29	MN259042	Canine parvovirus	Dog	Australia	Unpublished
30	OM100572	Canine parvovirus	Dog	India	Unpublished
31	LC646119	Canine parvovirus	Dog	India	Unpublished
32	LC646118	Canine parvovirus	Dog	India	Unpublished
33	MH576478	Feline panleukopenia virus	Cat	Thailand	Unpublished
34	MW091486	Feline panleukopenia virus	Giant panda	China	Unpublished
35	AJ002932	Feline panleukopenia virus	Modified live viral vaccine	Germany	Unpublished
36	KJ813893	Feline panleukopenia virus	Bobcat	USA	Unpublished
37	KP019620	Feline panleukopenia virus	Small Indian civet	Thailand	Unpublished
38	OM810195	Feline panleukopenia virus	Tiger	China	Unpublished
39	OM810192	Feline panleukopenia virus	Tiger	China	Unpublished
40	OM810194	Feline panleukopenia virus	Tiger	China	Unpublished
41	OM810197	Feline panleukopenia virus	Lion	China	Unpublished
42	MH127912	Feline panleukopenia virus	Cat	Taiwan	Unpublished
43	FJ405225	Feline panleukopenia virus	Tiger	China	Unpublished
44	MN722632	Foot-and-mouth disease virus	Cattle	Bangladesh	Unpublished

**Fig. 2:**
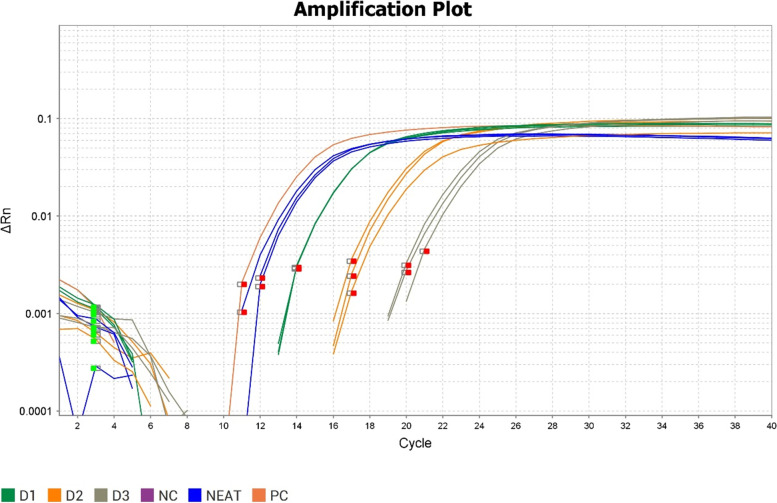
Amplification plot of query sample along with ten-fold dilution samples (D1 (1:10), D2 (1:100) and D3 (1:1000) along with neat (Undiluted) using CPV-2 as Positive control (PC) and Negative Control (NC) using SYBR assay

**Fig. 3:**
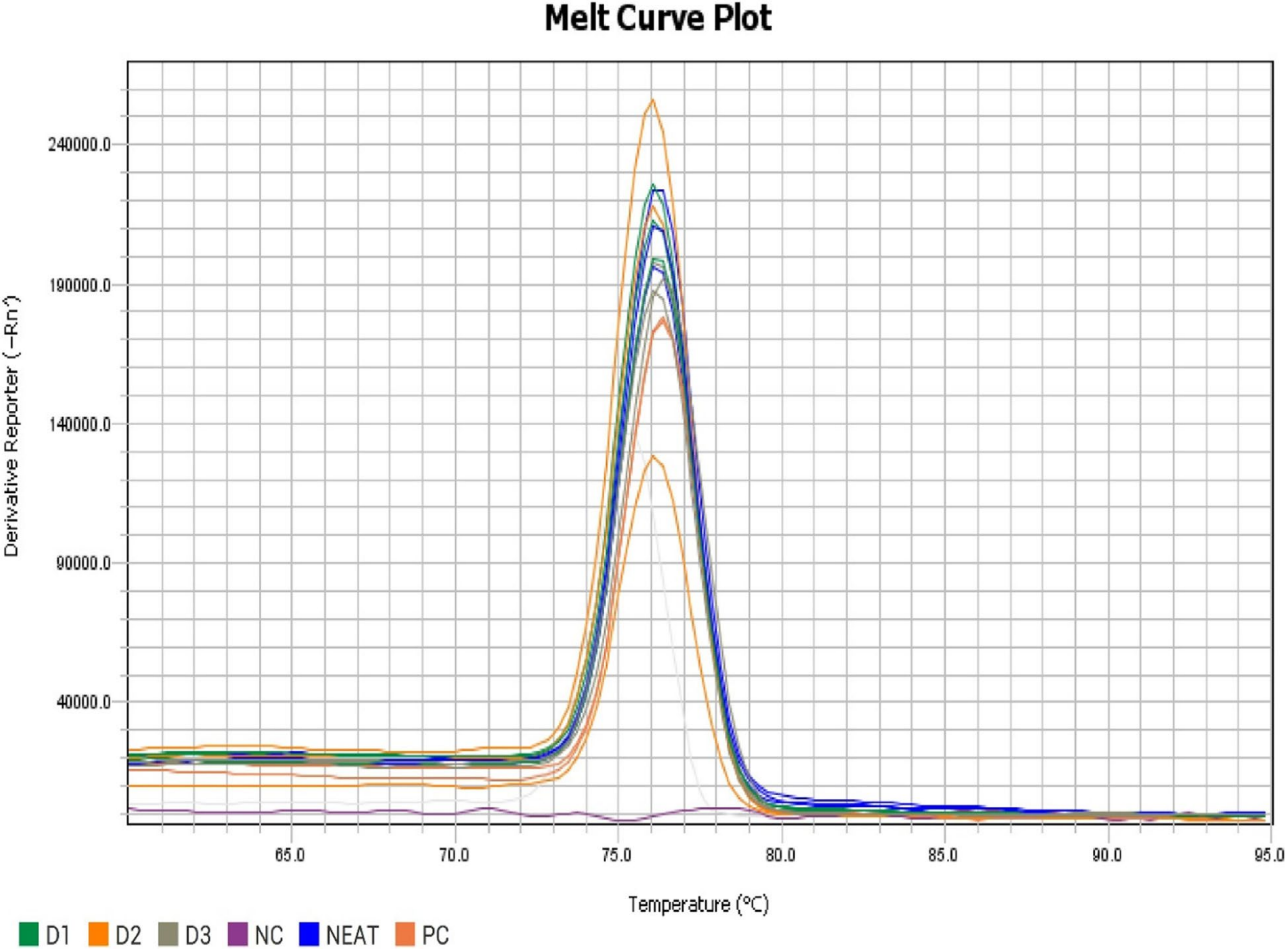
Melt curve plot of query sample along with ten-fold dilution samples (D1, D2 and D3) using SYBR assay

The PCR produced an amplification of approximately 680 bp, indicating a positive result. The qPCR provided an amplification corresponding to a mean CT value of 16.17 against a positive control with a mean CT value of 14.97 (Table 4) (Figs. 2 and 3). The amplicon was sequenced using the forward and reverse primers to ensure accuracy. On submission to the nBLAST tool of the National Center for Biotechnology Information (NCBI), the sequence was found to be 99.31% identical to isolates of FPLV reported in cats (Accession Nos. KT240134, MK671180, MF541127, AB0564227) similarly, the sequence had 99.14% similarity to FPLV reported in Racoons (Accession No. JN867594). The sequence was submitted to National Center for Biotechnology Information (NCBI) and allotted accession number ON129565.

**Table 4:** qPCR Results indicating the CT (mean), CT SD (Standard Deviation) and Tm values of the neat and diluted query sample

Sr. No	Sample	Dilution	CT (mean)	CT SD	Tm
1	Query Sample	Neat	16.17	0.26	76.22
2	D1	1: 100	17.67	0.01	76.07
3	D2	1: 1000	21.12	0.75	76.07
4	D3	1:10000	23.98	0.36	76.22
5	Positive control	Neat	14.97	0.01	76.36
6	Negative Control	NA	32.33	0.04	––––

**Fig. 4:**
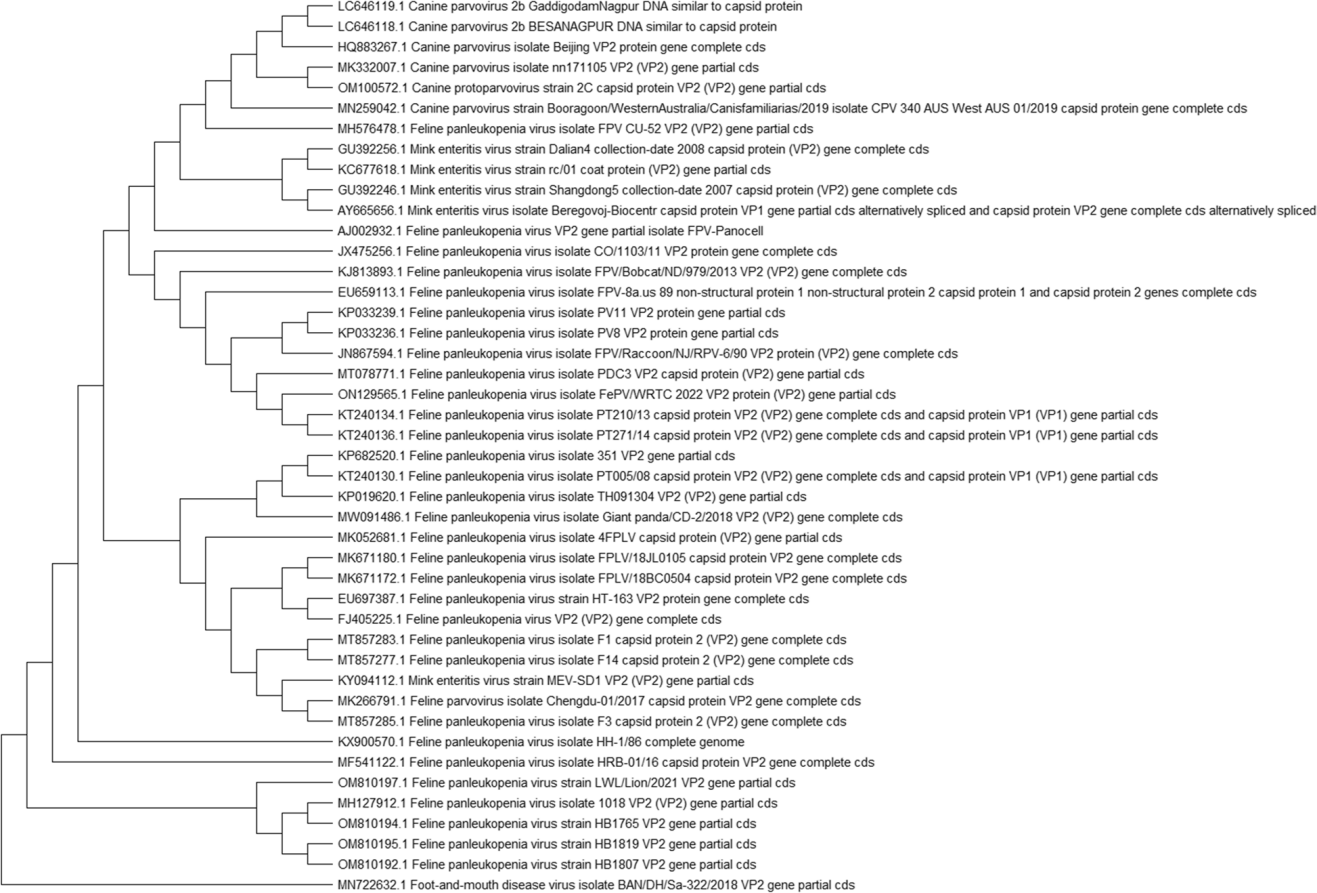
Phylogenetic analysis of sequences by maximum likelihood method using bootstrap method (1000 replications) to ensure tree consistency

To obtain greater understanding, the Maximum likelihood approach based on Tamura-Nei model was used to construct a phylogenetic tree using Mega XI software. The phylogenetic tree consisted of three distinct clades, with clade one consisting of CPV-2, MPV and FPLV sequences which maintained interclade identity under subclade IA, IB and IC respectively. The query sequence was placed in subclade IC and showed close similarity with the sequences of FPLV sequences reported from South Africa in cheetah (KP033239, KP033236), Portugal in cats (KT240134, KT240136) and USA in racoon (JN867594). However, sequences reported in tigers from China were separately placed in clade III (OM810192, OM810194, OM810197). The Foot and Mouth Disease Virus (FMDV) formed a consistent outgroup (Fig. 4).

## Discussion and conclusion

India is one of the seventeen mega-diversity hotspots and it is home to 3/4^th^ of the world's tigers. The native fauna of India includes important felid species like the asiatic lion (*Panthera leo*), royal bengal tiger (*Panthera tigris*), leopard, and clouded leopard (*Neofelis nebulosa*), which are all endangered due to lack of habitat, human-wild conflict, poaching, illegal trade and diseases. Leopards are protected under schedule I of the Wildlife Protection Act, 1972, which is the highest level of protection. The leopards were once distributed throughout the country; however, today, a few endemic populations survive distantly separated from each other. FPLV is known to cause considerable mortality in young cubs; orphan cubs are separated from their dams at an early stage of life. The passive immunity acquired on account of lactation is compromised due to the non-availability of natural lactation; such animals are more susceptible to FPLV in early life. FPLV and Canine Parvovirus (CPV-2) have been routinely treated in domestic cats and dogs worldwide. Treatment regimens consisting of aggressive fluid therapy, haemostatics, supportive vitamins and antibiotics have been successfully used with success in domestic animals. Considering the inappetence, dehydration due to vomition and diarrhoea and the persistent hot-dry weather, fluids were administered twice daily to ensure apt hydration. The animal was treated with standard supportive therapy to hasten recovery. The recombinant human granulocyte colony stimulating factor (rHGSF) has been used with success in the treatment of FPLV in cats [26], without any significant side effects. The FPLV infection is characterized by steep drop in the leucocyte count that can be effectively corrected by using rHGSF [27, 28]. The rHGSF is a glycoprotein that stimulates bone marrow and produce granulocytes, the majority of the case fatalities in FPLV are due to secondary bacterial infection due to resulting leukopenia [29, 30].

The first serological evidence of circulating viruses among wild felids was reported in 1991 [31]. However, with molecular tools and evolutionary analysis, new critical information has emerged that can be utilized better to understand the evolutionary relationship and emergence of viruses. The qualitative epidemiological investigation based on phylogenetic analysis helped understand the epidemiology of the disease in the landscape. In the current study, phylogenetic analysis helped in understanding the close evolutionary linkages between the CPV-2 and FPLV. The FPLV is considered as an ancestor of the CPV-2 [32] and the high degree of similarity in the VP2 region among viruses reported from a diverse geographical origins and hosts has been clearly demarcated in the study as previously reported [33]. The phylogenetic analysis by the Maximum Likelihood Method clearly pointed out the close similarity among the sequences of FPLV reported in cheetah from South Africa, racoons from USA, cats from Portugal. However, the sequences reported in Tigers from China was placed in a distinct clade. Since there are very few reports of FPLV in wild felids, it is crucial to undertake molecular investigations into such isolated incidences to gather epidemiologically substantial data on the circulating strains of the viruses. Many zoos and rescue centres engaged in wildlife conservation utilize vaccines developed for domestic cats due to the unavailability of vaccine strains from wild animals. The study also has marked the distinctness of the FPLV isolates from wild and domestic cats. Thus, if supplemented with molecular and phylogenetic studies, isolated studies can help generate data on the epidemiology of the circulating strains of viruses in the region.

Many large and medium felids are endangered, and viral infections can significantly hamper the conservation efforts directed to save the species from extinction. There are few reports on the impact of the FPLV on young cubs of large carnivores. Also, very few protocols for treating the treatment of large felids infected with FPLV are available. Diagnosis of FPLV is currently utilizing PCR and qPCR technologies for faster and more sensitive detection of the virus from the clinical samples. However, investigation regarding the circulating strains and other epidemiology attributes in the wild is still naive. An attempt to understand the epidemiological aspect of the circulating strains of FPLV has been made in the report. The findings have implication in deciding the protocol for treatment of wild cats infected with FPLV.

## Data Availability

The sequence identified in the study is available in the public domain database of NCBI under Accession No. ON129565. https://www.ncbi.nlm.nih.gov/nuccore/on129565.

## Abbreviations

FPLV: Feline Panleukopenia Virus
PCR: Polymerase Chain Reaction
NCBI: National Center for Biotechnology Information
MPV: Mink Parvovirus
CPV-2: Canine Parvovirus
DNA: Deoxyribonucleic Acid
TCID: Tissue Culture Infectious Dose
rHGSF: Recombinant Human Granulocyte Colony Stimulating Factor
FMDV: Foot and Mouth Disease Virus
CCTV: Closed Circuit Television
